# Discovery and structural investigation of Varicella-Zoster virus gE-neutralizing antibodies isolated from a convalescent patient

**DOI:** 10.1093/procel/pwaf051

**Published:** 2025-06-30

**Authors:** Lulu Wang, Zihan Jia, Xiaohan Ye, Chunxiao Chen, Baofa Sun, Xiangshuai Zhao, Ruiqi Zhang, Ying Li, Wenya Wang, Zixian Sun, Lushuai Zhou, Zhiyu Ni, Nan Zhang, Yu Guo

**Affiliations:** State Key Laboratory of Medicinal Chemical Biology and College of Life Sciences, Nankai University, Tianjin 300071, China; Affiliated Hospital of Hebei Engineering University, Hebei University of Engineering, Handan 056002, China; Guangzhou National Laboratory, Guangzhou International Bio Island, Guangzhou 510005, China; Central Laboratory, Hebei Collaborative Innovation Center of Tumor Microecological Metabolism Regulation, Affiliated Hospital of Hebei University, Baoding 071000, China; State Key Laboratory of Medicinal Chemical Biology and College of Life Sciences, Nankai University, Tianjin 300071, China; State Key Laboratory of Medicinal Chemical Biology and College of Life Sciences, Nankai University, Tianjin 300071, China; State Key Laboratory of Medicinal Chemical Biology and College of Life Sciences, Nankai University, Tianjin 300071, China; State Key Laboratory of Medicinal Chemical Biology and College of Life Sciences, Nankai University, Tianjin 300071, China; State Key Laboratory of Medicinal Chemical Biology and College of Life Sciences, Nankai University, Tianjin 300071, China; State Key Laboratory of Medicinal Chemical Biology and College of Life Sciences, Nankai University, Tianjin 300071, China; Tianjin Second People’s Hospital, Tianjin 300192, China; Central Laboratory, Hebei Collaborative Innovation Center of Tumor Microecological Metabolism Regulation, Affiliated Hospital of Hebei University, Baoding 071000, China; Guangzhou National Laboratory, Guangzhou International Bio Island, Guangzhou 510005, China; State Key Laboratory of Medicinal Chemical Biology and College of Life Sciences, Nankai University, Tianjin 300071, China; Affiliated Hospital of Hebei Engineering University, Hebei University of Engineering, Handan 056002, China; Central Laboratory, Hebei Collaborative Innovation Center of Tumor Microecological Metabolism Regulation, Affiliated Hospital of Hebei University, Baoding 071000, China; State Key Laboratory of Medicinal Chemical Biology and College of Life Sciences, Nankai University, Tianjin 300071, China; State Key Laboratory of Medicinal Chemical Biology and College of Life Sciences, Nankai University, Tianjin 300071, China; Guangzhou National Laboratory, Guangzhou International Bio Island, Guangzhou 510005, China; Central Laboratory, Hebei Collaborative Innovation Center of Tumor Microecological Metabolism Regulation, Affiliated Hospital of Hebei University, Baoding 071000, China; Frontiers Science Center for New Organic Matter, Nankai University, Tianjin 300071, China

**Dear Editor,**

Varicella-zoster virus (VZV) is a highly contagious human herpesvirus that causes chickenpox upon primary infection and can later reactivate to cause shingles (Johnson and Rice, 2014). It is globally widespread, and more than 90% of individuals are infected by adolescence (Gershon et al., 2015). Varicella-zoster immune globulin (VZIG), containing high levels of VZV-specific antibodies, provides protection against varicella in high-risk individuals when administered postexposure, reducing infection risk and mitigating severe cases. However, studies on the structural basis of the effects of neutralizing antibodies (nAbs) against natural herpes zoster (HZ) infection remain elusive (Duchon et al., 2020; Gans and Chemaly, 2021).

VZV encodes various glycoproteins, such as glycoprotein B (gB), glycoprotein C (gC), glycoprotein E (gE), glycoprotein H (gH), glycoprotein I (gI), glycoprotein K (gK), and glycoprotein L (gL), which play critical roles in viral maturation and packaging (Zerboni et al., 2014). Among these glycoproteins, gE plays a crucial role in VZV viral infection, serving as the most antigenically potent and abundant glycoprotein present on both the viral envelope and the membranes of infected cells (Zerboni et al., 2011). gE is currently the sole antigen in recombinant herpes zoster vaccines and has relatively high protective efficacy (Fowler et al., 1995). However, its structure remains poorly understood, rendering it difficult to analyze its main mechanisms of action and further optimize vaccine antigens at the molecular level.

In this study, we used gE with different tags (gE-Avi and gE-Flag) as bait to isolate gE-specific antibodies from peripheral blood mononuclear cells (PBMCs) of a convalescent patient. Initially, B cells were enriched using anti-CD19 magnetic beads. Subsequently, gE-specific memory B cells were identified via flow cytometry using fluorophore-conjugated gE-Avi (BV421) and gE-Flag (PE), along with the memory B cell marker CD27 (Fig. 1A). A total of 18,000 cells were processed for single-cell transcriptome and VDJ sequencing. UMAP analysis classified B cells into naïve, intermediate, memory, and plasma cells (Figs. 1B and S1A). Naïve B cells were rare, while memory and intermediate B cells exhibited distinct marker expression (Fig. 1C). To refine nAb identification, IgG1-expressing B cells with stable responses and a somatic hypermutation rate higher than 2% were selected (Fig. 1D) (Cao et al., 2020), yielding 100 enriched clonotypes (Fig. S1B). Further analysis of the 100 selected sequences revealed that most of the cells appeared at a low frequency, and the SHM rates were mostly between 0.15 and 0.2, indicating that most B cells had undergone affinity maturation (Fig. 1E). VDJ analysis revealed frequent recombination of *IGHV3-33/IGHV2-5/IGHV1-3/IGHV5-51* (heavy chains) and *IGLV3-21/IGLV3-14/IGKV1-39* (light chains) in naturally infected individuals (Fig. 1F).

**Figure 1.**
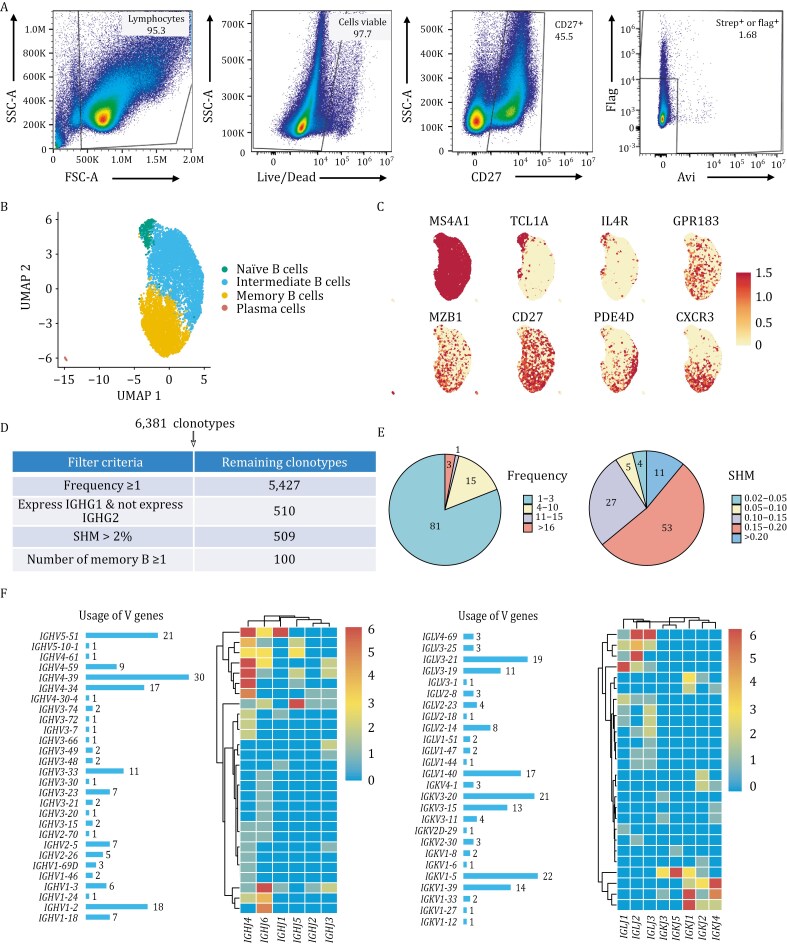
Isolation of gE-specific mAbs. (A) Strategy for sorting gE-specific B cells from convalescent PBMCs. Flow cytometry analysis was performed using CD27, gE-Avi, and gE-Flag markers, with Brilliant Violet 421 anti-human CD4 and PE-conjugated anti-human CD4 antibodies used as single-stain controls for gating. (B) The UMAP representation of sorted B cells. These cells can be classified into naïve B cells, intermediate B cells, memory B cells, and plasma cells based on markers. Memory B cells are characterized by high expression of CD27, GPR183, CXCR3, and PDE4D. (C) Expression levels of different cell surface markers. (D) Selection criteria for antibodies with high confidence. (E) Frequency of candidate antibodies, the number of memory B cells, and the distribution of SHM. (F) VDJ gene rearrangement of high-confidence candidate heavy and light chains.

We screened 53 ideal candidates with distinct CDR regions and high confidence, then conducted germline phylogenetic analysis to examine antibody responses to gE after infection (Fig. S2). The binding activity of each monoclonal antibody (mAb) to gE was evaluated using an enzyme-linked immunosorbent assay (ELISA). Among the 53 sequences, 7 elite antibodies exhibited high binding activity, with EC_50_ values ranging from 0.1 to 0.3 nmol/L (Figs. 2A and S3A). The binding affinities of these 7 elite antibodies were further analyzed by surface plasmon resonance (SPR), which revealed that all 7 elite antibodies had strong affinity for gE (Fig. S3B–H).

**Figure 2.**
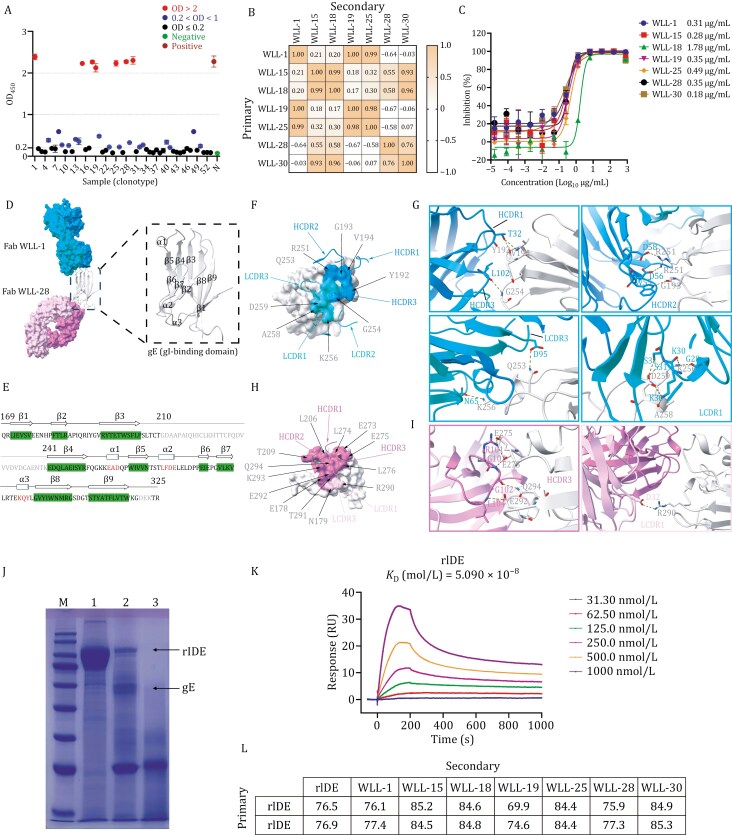
Identification and characterization of nAbs and epitope analysis of antigen-antibody complexes. (A) Identification of the binding activity of 53 mAbs. The binding activity of the antibodies to gE was evaluated by ELISA. Based on the OD_450_ results, the antibodies were classified as nonbinding, weakly binding, or strongly binding. Antibodies with strong binding activity are marked in red, whereas weak-binding and nonbinding antibodies are represented in blue and black, respectively. Selected weak-binding and strongly binding antibodies were subjected to serial dilutions to calculate the EC_50_, as shown in Fig. S3. (B) A competitive ELISA was used to detect the cross-competition of antibodies. (C) Identification of the neutralizing activity of strongly bound antibodies. The *Y*-axis indicates the ability of the antibodies to inhibit viral infection. (D) Cryo-EM structure of the ternary complex Fab WLL-1/Fab WLL-28 and the gI-binding domain of gE. The specific details of the gI-binding domain are displayed in the enlarged panel on the right. The gI-binding domain is colored silver, with Fab WLL-1 heavy and light chains colored dodger blue and dark turquoise and Fab WLL-28 heavy and light chains colored medium orchid and plum, respectively. (E) Sequence information and 2D structure of the gI-binding domain (aa 169–325). Visible residues in the electron density map are shown in black, invisible residues are shown in gray, the β-strand is shown in green background, and the α-helix is shown in red font. (F) The main residues involved in the interaction with the gI-binding domain of gE. gE is shown as a surface representation, and the others are shown in ribbon diagram surface representations. (G) Interface contact residues of the gI-binding domain (silver) and Fab WLL-1 heavy chain (dodger blue) and light chain (dark turquoise). Hydrogen bonds are shown as short gray sticks. (H) CDRs are involved mainly in interactions with the gI-binding domain. (I) Interface contact residues of the gI-binding domain (silver) and Fab WLL-1 heavy chain (medium orchid) and light chain (plum). Hydrogen bonds are shown as short blue sticks. (J) Pull-down assay validating the interaction between gE and rIDE. Lane 1: purified rIDE receptor protein. Lane 2: Sample of receptor protein captured after gE-Flag protein was immobilized on Flag resin. Lane 3: Sample after incubation of rIDE with Flag resin. (K) SPR determination of the dissociation constant of gE to rIDE. (L) Blocking effect of different antibodies on rIDE. The gE protein was presaturated with an excess amount of antibody, and rIDE was used to assess the competitive interaction between rIDE and the antibody. The results are expressed as a percentage of competition.

Epitope competition ELISA categorized the 7 elite antibodies into 3 groups, revealing at least 3 distinct gE antigenic sites (Fig. 2B). *In vitro* neutralization assays showed all 7 antibodies blocked VZV infection, with activities ranging from 0.1 to 2 µg/mL (Fig. 2C). Further germline analysis revealed that first class antibodies shared IGLV3-21 light chains with IGHV2-5/IGHV2-70 heavy chains. The second class included IGHV3-33, IGHV3-15, and IGHV1-3 heavy chains with IGLV2-14, IGKV2-30, and IGLV1-47 light chains. The third class had a single antibody with an IGHV5-51 heavy chain and an IGKV3-15 light chain. Compared with gE-specific antibodies elicited by the live-attenuated zoster vaccine (ZVL) (Sullivan et al., 2018), IGHV4-39 (heavy) and IGKV1-5 (light) showed weak binding in natural infection but strong binding postvaccination. Interestingly, further analysis of the BCR results revealed that, in contrast to the lack of IgM antibody-secreting cells 7 days after vaccination, IgM was still highly present in the B cells of the convalescent patient, suggesting that low-level persistent infection might still exist in the patient’s body (Fig. S1B). The heavy and light chains encoded by these genes are frequently involved in VDJ recombination, and the SHM rates of the 3 classes of antibodies were all greater than 0.2, indicating that each of the 7 elite nAbs had undergone moderate affinity maturation (Fig. S4).

We then used cryo-EM to analyze gE complexes with noncompetitive antibodies. A ternary complex (Fab WLL-1/WLL-28/gE) was constructed (Fig. S5A) and confirmed to be homogeneous and well-dispersed through negative-staining electron microscopy (Fig. S5B and S5C). Cryo-EM analysis resolved the Fab WLL-1/WLL-28/gE complex at 3.36 Å (Fig. S6; Table S1), revealing the binding sites of Fab WLL-1 and Fab WLL-28 on gE. The overall structure of the gI-binding domain of gE and Fab WLL-1/WLL-28 complex resembles a V-shape, with the 2 antibodies binding on opposite sides of the gI-binding domain of gE (Fig. 2D). The gI-binding domain of gE consists of 9 β-strands (β1–β9) and 3 α-helices (α1–α3), forming an IgG-like fold (Fig. 2E). Structural analysis revealed that the β-strands can be divided into 2 clusters. One cluster forms a β-sheet comprising β1, β3, β4, β5, β8, and β9, whereas the other cluster is formed by β2, β6, and β7 (Fig. 2E). Notably, the cryo-EM density for residues 210–241 is ambiguous, and this region is speculated to correspond to the gI binding site (Harshbarger et al., 2024). The electron density was also not observed in this region of the recently resolved gE crystal structure, indicating that gE is highly flexible (Harshbarger et al., 2024).

The structure reveals that Fab WLL-1 binds to one side of the gI-binding domain, with a buried surface area (BSA) of 683 Å^2^. Both the heavy and light chains of Fab WLL-1 contribute to this interaction, with 55% of the BSA derived from the heavy chain and 45% from the light chain (Fig. S7A; Tables S2 and S3). Fab WLL-1 mainly interacts with a loop formed by residues 191–195, situated between β2 and β3, as well as another loop encompassing the α1 helix formed by residues 251–261 (Fig. 2F). The complementarity-determining regions (CDRs) H1, H2, H3, L1, L2, and L3 of Fab WLL-1 mediate the interaction with the gI-binding domain (Fig. 2G; Tables S4 and S5) (see Supplementary Materials). Fab WLL-28 binds to an opposite side of the gI-binding domain, noncompetitively with Fab WLL-1 (Fig. 2D). The BSA for the gI-binding domain/Fab WLL-28 interaction is 578 Å^2^. The CDRH1-H3, CDRL1, and CDRL3 of Fab WLL-28 contribute to the interaction with the gI-binding domain, with the major contribution coming from CDRH1-H3, accounting for a BSA of 538 Å^2^ (Fig. S7B; Table S2). The regions of the gI-binding domain interacting with Fab WLL-28 are primarily composed of loop regions, including residues 273–276 located between β5 and β6, a loop region containing the α3 helix formed by aa 290–294, loops between β1 and β2 (aa 178–179), and between β3 and β4 (aa 206–209) (Fig. 2H; Tables S6 and S7). Five hydrogen bonds and 3 salt bridges are formed between Fab WLL-28 and the gI-binding domain. All hydrogen bonds originate from CDRH3 and interact with E275, Q294, and E292 of the gI-binding domain (Fig. 2I). Three salt bridges are formed between R101, D57, and D32 from Fab WLL-28 and E275, K293, and R290 of the gI-binding domain (Table S8). Additionally, a hydrogen bond is formed between D32 of CDRL1 and R290 of the gI-binding domain (Fig. 2I).

VZV gE has a unique N-terminal region (aa 1–188) that binds the cellular receptor insulin-degrading enzyme (IDE) and has been identified as immunodominant, underscoring its significance in the antibody response (Fowler et al., 1995; Garcia-Valcarcel et al., 1997; Zhu et al., 2016). In this study, we isolated nAbs targeting this region from the peripheral blood of a convalescent patient—an observation not previously reported for vaccine-induced antibodies. Epitope analysis revealed 2 main binding sites: the N-terminal domain (aa 1–188) (see Supplementary Materials), corresponding to the receptor-binding region, and the gI-binding domain.

To investigate the mechanism of neutralization, we expressed recombinant soluble IDE (rIDE, residues 42–1,019) and performed binding assays. A pull-down assay using gE immobilized on anti-Flag affinity resin confirmed a direct interaction with rIDE (Fig. 2J). SPR analysis further revealed a moderate binding affinity, with a *K*_D_ of ~10^−8^ mol/L (Fig. 2K), reinforcing the role of rIDE as a functional receptor for gE.

We then examined whether the nAbs interfere with this interaction. gE was pre-incubated with each antibody to form complexes, which were tested for rIDE binding using ELISA. Despite recognizing distinct epitopes, all nAbs effectively blocked the gE-rIDE interaction (Fig. 2L), suggesting that their neutralizing activity is primarily mediated through interference with receptor engagement.

Recent advances in structural biology have revolutionized our understanding of viral mechanisms and accelerated the development of antiviral therapies (Guo et al., 2023; Oliver et al., 2020; Wang et al., 2019). This study is the first to report the discovery and structural investigation of nAbs from convalescent patients and to compare the differences in antibody binding epitopes between individuals who recovered from natural infection and those vaccinated with ZVL. In the final stage of our manuscript preparation, Wayne D. Harshbarger et al. achieved important breakthroughs in the structural investigation of gE antibodies isolated from vaccine recipients. They used Fab fragments of anti-gE antibodies previously isolated from ZVL vaccine recipients to stabilize gE and map the epitopes targeted by human anti-gE antibodies induced by ZVL vaccination. Preliminary characterization of the gE structure has been achieved by defining 3 primary neutralizing epitopes by vaccine-induced human antibodies against gE (Harshbarger et al., 2024). One epitope is located at the Fc-binding domain, which is involved in the interaction between gE and Fc. The other 2 epitopes are within the gI-binding domain. The first of these is a β-sheet cluster formed by β2, β6, and β7, which are recognized by several antibodies, including 1E3, 1E12, and 5B3. The second epitope is located in the loop region between β3, β4, and β9, where antibody 1A2 binds.

We report the identification and characterization of nAbs obtained from a convalescent patient. All antibodies examined bind to the gE protein (aa 1–325). Notably, WLL-1 and WLL-28 both bind within the gI-binding domain but differ in their binding sites. WLL-1 primarily interacts with the loop region between β2 and β3, as well as β4 and β5, overlapping partially with the binding sites of antibody 1A2. WLL-28 predominantly binds to the loop region between β5 and β6, β7 and β8, in close proximity to where antibodies 1E3 and 1E12 interact with the β-sheet cluster formed by β2, β6, and β7 (Fig. S8). Consistent with findings from antibodies isolated from patients after ZVL immunization, no antibodies specific to the gE–gI heterodimer interface residues (aa 208–236) were detected. This may be attributed to the formation of the gE–gI heterodimer, which likely prevents these gE residues from being exposed. These findings indicate that epitopes recognized by antibodies from natural infection may differ from those induced by vaccination. Furthermore, the fact that many of the neutralizing epitopes are located in the gI-binding domain suggests that this region shows potent immunogenicity, as well as the potential to elicit an effective antibody response, offering valuable insights for the development of next-generation recombinant vaccines.

## Supplementary data

Supplementary data is available at *Protein & Cell* online https://doi.org/10.1093/procel/pwaf051.

pwaf051_Supplementary_Data

## Footnotes

We thank Li X, Mi Y, Pang E, Li W, and Dong Q at the Cryo-EM Center, Shanxi Academy of Advanced Research and Innovation for their technical support on Cryo-EM.

This work was supported by the National Natural Science Foundation of China (Grant Nos. 32271256 and 32471263 to Y.G., 82373072 to Z.N., 32300146 to Z.S.), the Fundamental Research Funds for the Central Universities (030-63253192 to Y.G., 030-63241622 to N.Z.), the Major Project of Guangzhou National Laboratory (GZNL2023A01002 to Z.S.), the Natural Science Foundation of Hebei Province (H2022201067 to Z.N.), and the Central Government Guides Local Science and Technology Development Special Project (236Z7744G to Z.N.), the China Postdoctoral Science Foundation (2023M741824 to N.Z.), State Key Laboratory of Medicinal Chemical Biology (035-BB042511), the Funding Support for Key Research Teams (035-9251700302).

Y.G., L.W., Z.J., N.Z., X.Z., C.C., and B.S. are listed as inventors on pending patent application No. 202411184268.5 related to this work. All other authors declare no competing interests.

This study was approved by the Ethics Committee of the State Key Laboratory of Medicinal Chemical Biology at Nankai University (Ethics Approval Number: NKUIRB2023093). All sample collection and laboratory procedures were conducted in accordance with the approved protocols and relevant guidelines.

Written informed consent was obtained from the participant prior to blood sample collection and B cell isolation procedures.

The participant provided written consent for publication of anonymized data and findings derived from the sample used in this study.

The cryo-EM density maps and corresponding atomic coordinates of the Fab WLL-1/Fab WLL-28 and gI-binding domain of gE ternary complex have been deposited in the Electron Microscopy Data Bank (EMDB) and Protein Data Bank (PDB), respectively. The accession codes are: EMD-61939, PDB 9JZQ.

These authors jointly supervised this work: Zhiyu Ni, Nan Zhang, Yu Guo.

L.W., Z.J., X.Y., and Y.G. jointly designed the experiments, conducted the experiments, analyzed the data, and wrote the paper. C.C. and B.S. contributed to the bioinformatics analysis. R.Z., X.Z., Y.L., Z.S., L.Z., and W.W. each contributed to conducting the experiments and analyzing the data. Z.N., N.Z., and Y.G. supervised the work and assisted in writing the paper. All authors reviewed and accepted the manuscript.

This study did not generate new custom code. All analyses were performed using publicly available software as described in the Methods section.

AlphaFold, an AI-based protein structure prediction tool developed by DeepMind, was used to predict the structure of (target protein/domain) in this study. The AlphaFold Protein Structure Database or the AlphaFold2 server was used as appropriate.
