# Supplementary material for: Discovery and structural investigation of Varicella-Zoster virus gE-neutralizing antibodies isolated from a convalescent patient
Source: Protein Cell. 2025 Jun 30;16(12):1054–9. doi: 10.1093/procel/pwaf051 (PMC12742838; doi:10.1093/procel/pwaf051)
Supplement: pwaf051_Supplementary_Data [file pwaf051_supplementary_data.zip › pwaf051_suppl_Supplementary_Figures_S1-S8_Tables_S2-S8.pdf]

Supplementary Materials

Discovery and structural investigation of Varicella-Zoster virus gE-neutralizing antibodies isolated from a convalescent patient

Lulu Wang[1,2,3,4†], Zihan Jia[1†], Xiaohan Ye[1†], Chunxiao Chen[1], Baofa Sun[1], Xiangshuai Zhao[1], Ruiqi Zhang[1], Ying Li[5], Wenya Wang[4], Zixian Sun[3], Lushuai Zhou[1], Zhiyu Ni[2,4*], Nan Zhang[1*], Yu Guo[1,3,4,6*]

[1]State Key Laboratory of Medicinal Chemical Biology and College of Life Sciences, Nankai University, 38 Tongyan Road, Tianjin 300071, China.

[2]Affiliated Hospital of Hebei Engineering University, Handan, Hebei 056002, China.

[3]Guangzhou National Laboratory, No.9 Xing Dao Huan Bei Road, Guangzhou International Bio Island, Guangzhou 510005, Guangdong Province, China.

[4]Central Laboratory, Hebei Collaborative Innovation Center of Tumor Microecological Metabolism Regulation, Affiliated Hospital of Hebei University, Baoding, Hebei 071000, China.

[5]Tianjin Second People's Hospital, Nankai, Tianjin 300192, China.

[6]Frontiers Science Center for New Organic Matter, Nankai University, China.

[†] These authors contribute equally to this work: L.W, Z.J, X.Y

*Correspondence should be sent to: Y.G (guoyu@nankai.edu.cn), N.Z (zhangnan@nankai.edu.cn), and Z.N (nizhiyu@hebeu.edu.cn).

[†]These authors contributed equally to this work.

Contents

Supplementary Information and Methods

Supplementary Figures

Supplementary Tables

References

Supplementary Information and Methods

Cryo-EM structure of the complex between gE and the third group Fab

To analyze the binding mechanisms of the antibodies to gE, we constructed the quaternary complex of Fab WLL-1/Fab WLL-15/WLL-28 and the full-length extracellular domain of gE for cryo-EM analysis (Fig. S5a). The specific states of these complexes were assessed using negative-staining electron microscopy. The results revealed that complex was homogeneous and well dispersed (Fig. S5b and S5c). However, only the ternary complex structure of VZV gE with Fab WLL-1 and Fab WLL-28 was obtained, which included a portion of gE along with the two Fab antibodies (Fig. S5d and S5e). Efforts were also made to resolve the cryo-EM structure of the full-length extracellular domain of gE in complex with Fab WLL-15. Although the presence of the antibody was observed in the 2D classification, the antigen density remained ambiguous, preventing the generation of a high-resolution complex structure (Fig. S5f). The ELISA results indicated th at WLL-15 binds to the aa 1-169 region of gE, which corresponds to a long loop in the predicted structure. To further investigate the specific binding site of WLL-15, two truncated gE variants were generated and subsequently analyzed using ELISAs. Ultimately, WLL-15 binds to the aa 130-169 region of gE, suggesting that the high flexibility of this region may contribute to challenges in resolving its structure (Fig. S5g and S5h).

Structural details of the Fab WLL-1 and gI-binding domain

The Fab WLL-1 heavy chain forms six hydrogen bonds with the gI-binding domain, involving T32 and L102 of CDRH1 interacting with V194 and G254 of the gI-binding

domain and D58, D56, and W55 of CDRH2 interacting with R251 and G193 of the gI-binding domain. Additionally, three salt bridges are formed between D56 and D58 of the heavy chain and R251 of the gI-binding domain. The light chain forms eight hydrogen bonds: K30, G28, and S31 of CDRL1 interact with K256, A258, and D259 of the gI-binding domain, whereas D95 and N65 of CDRL3 interact with Q253 and K256 of the gI-binding domain. Furthermore, two salt bridges can be observed between K30 and D50 of the light chain and D259 and K256 of the gI-binding domain (Table S5).

Methods

Plasmid construction, antigen expression, and purification

The full extracellular domain of gE (aa 1-538) (GenBank accession no. Q9J3M8) was codon-optimized and cloned into the pCDNA3.1 vector. Flag-His and Avi-His tags were added separately at the C-terminal to construct pCDNA3.1-gE-Flag-His and pCDNA3.1-gE-Avi-His plasmids, which were used for subsequent expression.

Based on the predicted gE structure from AlphaFold Server 3 (Abramson et al., 2024) and previous literature, different truncations of gE were constructed: gE (aa 1-325), gI-binding domain (aa 169-325), and gE (aa 339-507). 8×His tag was added to the C-terminal of each truncated construct for subsequent purification.

rIDE is constructed from position 42 of the IDE human open reading frame, which is the second ATG, as the starting site for translation (GenBank access no. P14735). The codon-optimized rIDE was cloned into the pCDNA3.1 vector with an 8× His tag added at the C- terminal for purification purposes.

According to the manufacturer's instructions, the plasmids were transiently transfected into 293F cells using the transfection reagent PEI 25000 (Polyethylenimine Linear, MW 25000) (MKBio, cat#MX2202) at a ratio of 1:3. Approximately 5 days post-transfection, the supernatant was collected and incubated with High Affinity Ni-NTA Resin (GenScript, cat# L00250) at 4°C for 2 hours. Non-specific proteins were washed away with a wash buffer (25mM Tris, 150mM NaCl, 10mM imidazole, pH 7.5), and the target protein was eluted using an elution buffer (25mM Tris, 150mM NaCl, 500mM imidazole, pH 7.5). The eluted solution was concentrated using an Amicon Ultra centrifugal filter device (30 kDa MWCO). The target protein was then further purified into the buffer (25mM Tris, 150mM NaCl, pH 7.5) using Superdex 75 Increase 10/300GL or Superdex 200 Increase 10/300GL chromatography column in buffer (25 mM Tris, 150 mM NaCl, pH 7.5).

Isolation of PBMCs and sorting of specific B cells

The collected whole blood was used to isolate PBMCs through density gradient centrifugation using Human Peripheral Blood Lymphocyte Separation Medium (Solarbio, cat# P8610). An initial screening of B cells was conducted using a CD19 magnetic bead kit, and CD19-positive cell populations were selected for B cell enrichment according to the manufacturer's protocol (Miltenyi Biotec, cat#130-050-301). The gE-Avi tag protein was biotinylated following the manufacturer's instructions (Biotin-Protein Ligase/BirA Enzyme, cat #BI001, GeneCopoeia). The cells were first incubated with the anti-human Fc Receptor Binding Inhibitor at 4°C for 30 minutes (Invitrogen, cat#14-9161-73). The enriched cell population was then stained at 4°C for

30 minutes using Fixable Viability Stain 780 (BD Biosciences, cat #565388) and Brilliant Violet 650 anti-human CD27 antibody (Biolegend, cat#302828). After washing away excess dye, the stained cells were incubated with the biotinylated gE-Avi tag protein and the gE-Flag tag protein at 4°C for 30 minutes, followed by incubation with BV421 Streptavidin (BD Biosciences, cat#563259) and PE anti-DYKDDDDK Tag fluorescent dye (Biolegend, cat#637310) at 4°C for 30 minutes for flow cytometry analysis. Approximately 18,000 $CD19^+$, $CD27^+$, $gE\text{-}Avi^+$, and $gE\text{-}Flag^+$ antigen-specific binding B cells, with a viability of 97.5%, were sorted into the sequencing buffer.

Single-cell sequencing and analysis

As described previously (Guo et al., 2023), the sorted B cells were washed and resuspended to prepare a single-cell suspension, adjusted to an appropriate concentration of 700-1200 cells/μl, in preparation for sequencing. The sample was then loaded onto the 10× Chromium Next GEM Chip K (1000287), and the Chromium TM system was run to form single-cell micro-reaction systems, GEMs (Gel Bead in Emulsion), encapsulated by oil droplets. Recovery Agent was added to mix with the GEMs to break the oil layer, releasing cDNA. The cDNA was then amplified, purified, and enriched. The concentration of cDNA was measured using Qubit quantitation reagents to meet the requirements for library preparation. Sequencing was performed using the Novoseq 6000 platform in PE150 mode to obtain 5' transcriptome data and immune repertoire data from each cell.

CellRanger (v7.1.0) was used to process raw data, demultiplex cellular barcodes,

map reads to the human genome (GRCh38) and generate digital gene expression matrices; 8772 single cells from were detected. Less prevalent genes (<3) and cells with less prevalent genes (<200) were removed by Seurat (v4.3.1). Low-quality cells were removed if the number of expressed genes was <200 or >4,000. Cells were also removed if their proportion of mitochondrial gene expression was >3%. The top 1,500 variable genes identified by the 'vst' method were used in principal component analysis; 1-10 principal components (PCs) were used in the FindNeighbors function(Hao et al., 2021). The FindClusters function was used to identify clusters (res = 0.1), which were then visualized with two-dimensional UMAP plots. Marker genes for each cluster were identified by the "FindAllMarkers" function with the cutoff of adjusted P-values < 0.05 and $|logFC| > 0.25$. DoubletFinder (v.2.0) using an anticipated doublet rate of 6%, which is the expected rate reported by 10x Genomics for the number of cells loaded onto the 10x Controller (McGinnis et al., 2019). The remaining 8236 single cells were used in downstream analyses. Canonical marker genes were used to identify cell types. Combining the annotations of subtypes referring to with highly expressed signature genes (CD27, GPR183 and MZB1), Memory B cells were precisely identified for subsequent clonotype selection.

The BCR contig sequences were assembled and annotated using the "vdj" function of Cellranger (v7.1.0) with the GRCh38 reference genome (Guo et al., 2023). Only contigs labeled as high-confidence and productive were retained for clonotype analysis. Cells selected for further analysis required at least one heavy chain (IGH) and one light chain (either IGL or IGK). For cells with two or more assembled IGHs or IGLs/IGKs,

the dominant heavy or light chain was identified as the IGH or IGL/IGK sequence with the highest UMI count. The clonality degree of a given clonotype was assessed by the frequency of B cells sharing that clonotype. SHM was calculated as the total number of mismatches and gaps divided by the length of the heavy-chain VDJ DNA sequence. Mismatches and gaps were identified by aligning the heavy-chain VDJ DNA sequence to the germline sequence (sourced from the international ImMunoGeneTics information system (IMGT)) using Igblast (v1.22.0) (Ye et al., 2013). Heatmaps were created using the "pheatmap" package, while pie charts, bar plots, and boxplots were produced with the "ggplot2" package in R.

In vitro expression of antibodies and production of Fab fragments

The variable region genes of the heavy chains of the 53 antibody pairs, identified through bioinformatics analysis, were cloned into the pcDNA3.4 vector containing the human IgG1 constant region. and the light chain variable regions were ligated into the corresponding lambda or kappa pcDNA3.4 vectors to construct antibody expression plasmids. The recombinant plasmids containing the heavy and light chains from the same cell were co-transfected into 293F cells using the transfection reagent PEI 25000 at a ratio of 1:3. The cells were cultured under conditions of 5% CO_2, 37°C, with shaking at 120 rpm for 5 days. Afterward, 200 μL of culture supernatant was collected, and the binding activity to the VZV-gE was determined by ELISA. Antibodies with high binding activity were selected for gradient dilution to calculate their EC_{50} values (calculated based on the molecular weight of 144 kDa for the antibody, where 100 nM = 14.4 μg/mL). Antibodies with high binding activity were chosen for large-scale

expression and subsequently purified using Protein A medium (GenScript, cat#L00210). The purified antibodies were digested with papain at 37°C for 10 hours. The Fc fragments were captured using Protein A medium, and the flow-through containing the Fab fragments was collected. The Fab fragments were further concentrated using an Amicon Ultra centrifugal filter device (10 kDa MWCO).

Flag pull-down assay

For the Flag pull-down assay, gE and rIDE proteins were expressed in 293F cells. The gE-Flag-His and rIDE-His proteins were purified using High Affinity Ni-NTA Resin (GenScript, cat#L00250). Purified gE-Flag-His protein (100 μg) was incubated with 100 μL of anti-Flag affinity resin (GenScript, cat#L00432) on a flip shaker for 1 hour at 4 °C. After five washes with buffer (20 mM Tris-HCl, 150 mM NaCl, pH 7.5), rIDE-His protein was added to the resin and incubated on a flip shaker for 1 hour at 4 °C. The beads were then washed five additional times with the same buffer. Bound proteins were eluted by boiling in SDS loading buffer and analyzed by SDS-PAGE.

ELISA analysis of antibody binding activity

The VZV-gE-Flag-His protein was coated onto an ELISA plate at 2 μg/mL, 100 μL per well, and incubated overnight at 4°C. After washing, the plate was blocked with 5% skim milk (PBS). Then, 100 nM of mAb supernatant was added at 100 μL per well and incubated at 25°C for 1 hour. After washing the plate, a secondary antibody, Anti-human IgG Fc HRP (Abcam, cat#ab99759), was added at 100 μL per well and incubated at 25°C for 1 hour. A color development solution (Solarbio, cat#PR1200) was added at 100 μL per well for color development, followed by 50 μL per well of stop solution

(Solarbio, cat#C1058) before reading the OD_{450}. Wells with a colorimetric value above 0.5 were considered positive. Positive wells were further subjected to gradient dilution ELISA to calculate the EC_{50} value: Antigen coating and blocking were performed as described above. The antibody to be tested was initially diluted to 100 nM in the first well, followed by 4-fold serial dilutions across 8 gradients, 100 μL per well, and incubated at 25°C for 1 hour. After washing, the secondary antibody was added, followed by color development and reading to calculate the EC_{50}.

Antibody epitope competition ELISA

Antibody A was coated onto an ELISA plate at 0.2 μg/well, 100 μL/well, and incubated at 4°C overnight. The plate was then blocked with 5% skim milk at 37°C for 2 hours. Excess antibody B was mixed with gE and incubated for 1 hour, then the antigen-antibody mixture was added to the ELISA plate coated with antibody A and incubated for an additional hour. After washing, HRP-conjugated anti-Flag antibody (Proteintech, cat#HRP-66008) was added for detection. If color development occurs after incubation of excess antibody B with gE, it indicates that antibody B does not block the binding of antibody A to gE. Conversely, the absence of color indicates that binding is blocked.

mAbs binding affinity measurement

Surface plasmon resonance (SPR) technology (using Biacore T200) was employed to detect the binding kinetics and affinity between the antigen protein and isolated antibodies. Specifically, the antigen protein VZV-gE-His was immobilized as a ligand on a CM5 chip through amine coupling, using an amine coupling kit (Cytiva, cat#BR-1000-50). The coupling buffer was 10 mM sodium acetate at pH 4.0 (Cytiva, cat#BR-

1003-49). 7 elite antibodies were serially diluted in buffer (0.01 M HEPES, 0.15 M NaCl, 3 mM EDTA, 0.08% P20, pH 7.4) and used as analytes. The association time was set to 200 seconds, and the dissociation time was set to 1200 seconds, with one channel serving as a reference channel. The data were analyzed using Biacore Insight Evaluation Software, and the fitting model used was a 1:1 binding model.

Virus neutralization test

Human fetal lung fibroblasts MRC-5 (ATCC, cat#CCL-171) were seeded in a 96-well plate and incubated overnight in a cell incubator (37°C, 5% CO_2). The test antibody was diluted using diluent containing guinea pig complement (Solarbio, cat#S4990), starting at a concentration of 800 μg/mL, and then subjected to a 5-fold serial dilution across 12 dilutions. Diluted virus was added to the sample wells and virus control wells, followed by incubation in a 37°C, 5% CO_2 incubator for approximately 1 hour. The mixture was then added to MRC-5 cells, incubated for about 2 hours, and the medium was replaced with 100 μL of fresh medium per well for further incubation for approximately 48 hours. The supernatant was discarded, the cells were fixed, and a fluorescence-labeled detection antibody was added. The plate was read using a CTL S6 Ultra M2 instrument to calculate the NT_{50}.

Negative stain electron microscopy sample preparation

Add 4 μL of the purified WLL-1/WLL-28/gE and WLL-1/WLL-15/WLL-28/gE complex at a concentration 0.04 mg/mL onto a freshly glow-discharged carbon coated grid (300 mesh, Beijing Zhongjingkeyi Technology, China). The grid was allowed to incubate for 1 minute to facilitate sample adsorption. Excess sample was carefully

removed by blotting the grid with filter paper. The grid was then stained with 100 μL 3% (w/v) uranyl acetate for 30 seconds. After staining, the excess stain was carefully removed by blotting from one side, and the grid was left to air-dry at room temperature. The prepared grids were then loaded into a Talos L120C TEM (Thermo Fisher Scientific, USA), operated at an acceleration voltage of 120 kV.

Cryo-EM sample preparation and data collection

The purified complex was diluted to 0.3 mg/ml, and then 3.5 μl of the sample was added to a carbon Quantifoil Cu grid (R0.6/1, 300 mesh, Quantifoil, Micro Tools GmbH, Germany) with glow discharge (15 mA for 50 s) using a Vitrobot Mark IV (Thermo Fisher Scientific, USA). The grid was blotted for 4.5 s with a blot force of 0 and wait 5s at 4°C and 100% humidity, then rapidly frozen in liquid ethane.

Datasets were acquired using a 300kV Titan Krios G3i transmission electron microscope (Thermo Fisher Scientific, USA) equipped with a K3-subunit detector (Gatan, USA) under super-resolution mode at a nominal magnification of 105,000×, which yielded a pixel size of 0.69 Å. All data were automatically collected using EPU software (Thermo Fisher Scientific, USA). Each image was recorded as 32 frames, with a total electron dose was set to 60 e-/$Å^2$. The defocus range of each sample was -0.8 to -2.2 μm.

Cryo-EM data processing, model building and refinement

For the WLL-1/WLL-28/gE complex, a total of 4865 movie stacks were imported to CryoSPARC and processed using CryoSPARC software suite (Punjani et al., 2017). Initially, raw micrographs were motion-corrected and dose-weighted using the Patch

Motion Correction module. Contrast transfer function (CTF) parameters were estimated using the Patch CTF Estimation tool. After manual curation of micrographs to remove low-quality images, particle picking was performed using the Blob Picker. Approximately 2,156,098 particles were automatically picked, with a minimum particle diameter of 100 Å and a maximum particle diameter of 250 Å.

Extracted particles were subjected to multiple rounds of 2D classification to remove noise and poorly aligned particles. A total of 324,054 particles were ultimately selected for Ab-Initio reconstruction to generate an initial model. This model was further refined through homogeneous refinement to improve resolution and alignment. For datasets with sufficient particle quality and homogeneity, non-uniform refinement was employed to achieve high-resolution structures. The final resolution was determined to be 3.48 Å, based on the gold-standard Fourier shell correlation (FSC) criterion at 0.143. To enhance the interpretability of the density maps, automatic local sharpening was performed using deepEMhancer (Sanchez-Garcia et al., 2021), which significantly improved the clarity of structural features.

Using the AlphaFold3-predicted structures of gE and the two Fab fragments as models. Due to the presence of flexible regions in gE, only the density corresponding to the gI-binding domain was observed, while the Fc-binding domain was not resolved. Subsequently, the atomic models were fitted into the map using ChimeraX (Meng et al., 2023). The initial model was refined iteratively using real-space refinement in Phenix (Adams et al., 2010) to minimize geometric deviations and optimize the fit to the density map. Manual adjustments were made in Coot (Emsley et al., 2010), focusing

on regions with ambiguous density or side-chain placement. The figures were prepared using PyMOL (http://www.pymol.org) or UCSF ChimeraX.

Supplementary Figures

Figure S1

a

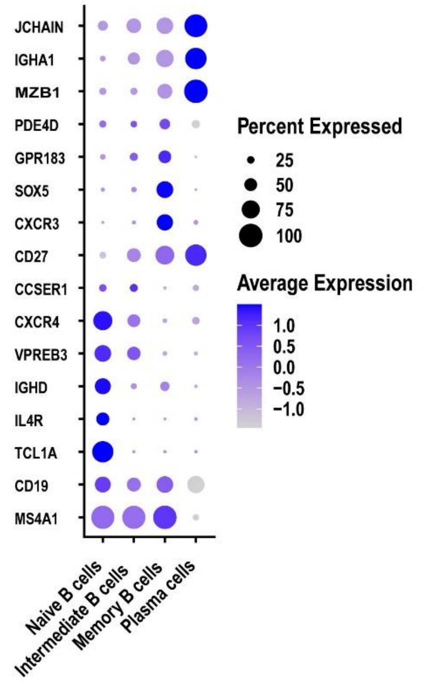

b

	Frequency>1	Frequency≥1
	IGHM+	IGHG1+
	IGHG2-	IGHG2-
	Memory B≥1	Memory B≥1
Cell number	1760	179
Clone type	718	136
Clone type (cellranger)		100

Fig. S1. Analysis of the sorted clonotypes by single-cell transcriptomics.
(**a**) Expression of different marker genes in the 8,236 clonotypes sorted. Cells can be divided into naïve B cells, intermediate B cells, memory B cells, and plasma cells on the basis of differences in highly expressed characteristic genes. (**b**) Classification of selected high-confidence clonotypes according to different criteria. The IGHM+IGHG2- B cells had 718 clonotypes, but the IGHG1+IGHG2- B cells had only 136 clonotypes. The sequences with only one chain were removed, leaving 100 correctly paired antibodies.

Figure S2

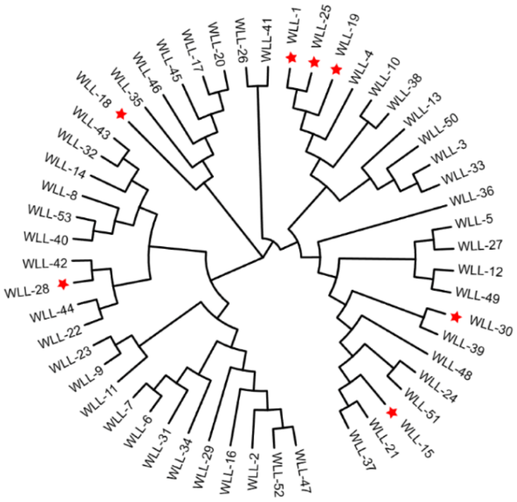

Fig. S2. A phylogenetic analysis of 53 antibodies was performed. Red asterisks indicate antibodies subsequently validated to have high neutralizing activity.

Figure S3

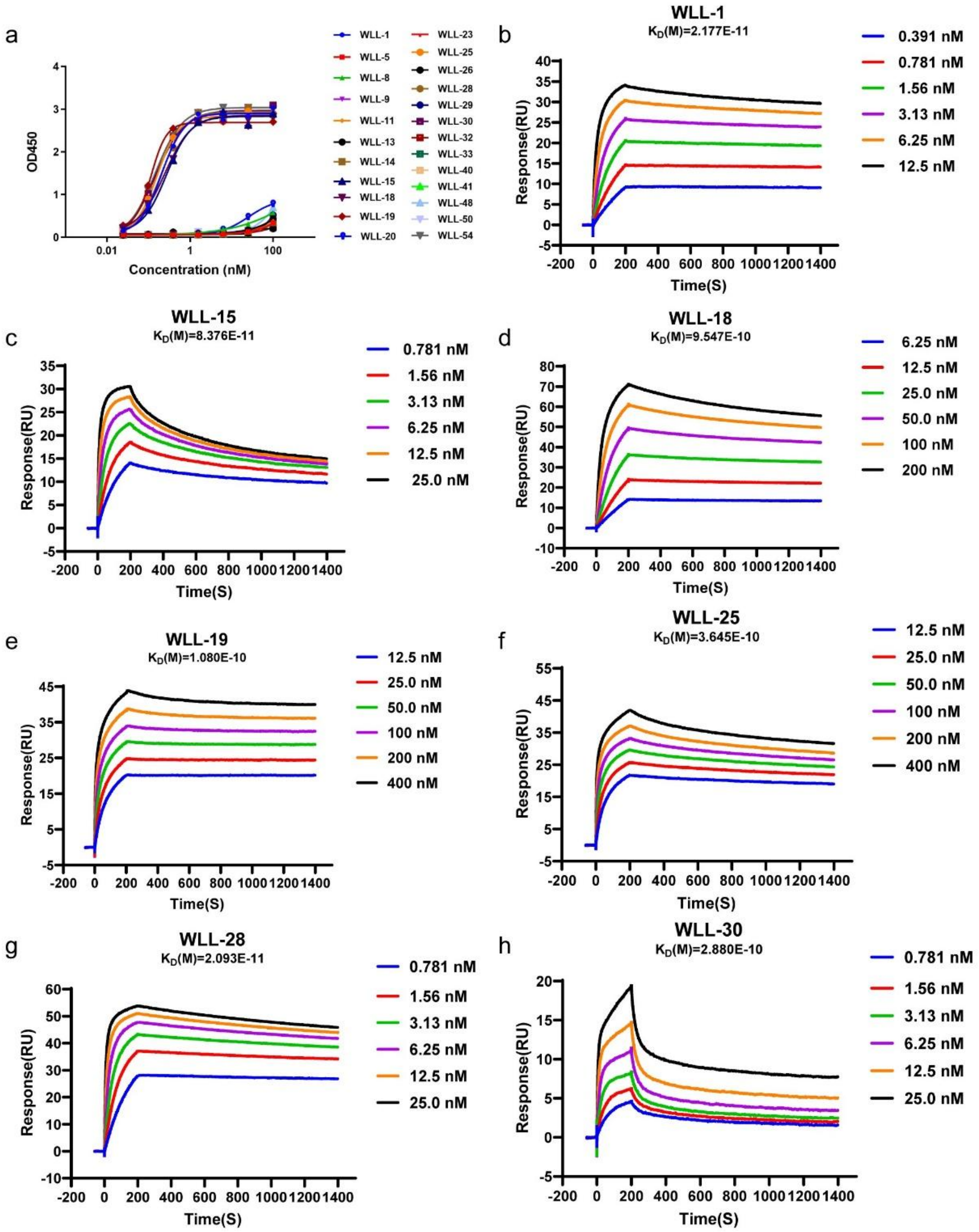

Fig. S3. Identification of mAb binding activity. (**a**) Antibody sorting and validation. Clones with an $OD_{450} > 2$ were selected for gradient dilution to calculate the EC_{50}. Consistent with previous experiments, 7 antibodies had high binding activity. (**b-h**) SPR determination of the dissociation constants of high-binding antibodies to gE.

Figure S4

Antibodies	Frequency	H-germline	L-germline	HCDR3	SHM	NT50(μg/ml)
WLL-1	19	IGHV2-5	IGLV3-21	CARLSLRLLTFDYW	0.582491582	0.31
WLL-19	3	IGHV2-5	IGLV3-21	CAPVCGPGTSGCWFDPW	0.771043771	0.35
WLL-25	2	IGHV2-70	IGLV3-21	CARTMYQFLGLDYW	0.567340067	0.49
WLL-15	4	IGHV3-33	IGLV2-14	CARGPSRLRDQLPALFMDVW	0.416666667	0.28
WLL-30	2	IGHV1-3	IGLV1-47	CASSFDHDNWGPGNHW	0.397435897	0.18
WLL-18	3	IGHV3-15	IGKV2-30	CATDVTGW	0.421130952	1.78
WLL-28	2	IGHV5-51	IGKV3-15	CARHVRGSLWGGGGYW	0.603896104	0.35

Fig. S4. Germline analysis of 7 elite antibodies. Different color shading indicates antibodies with different binding epitopes.

Figure S5

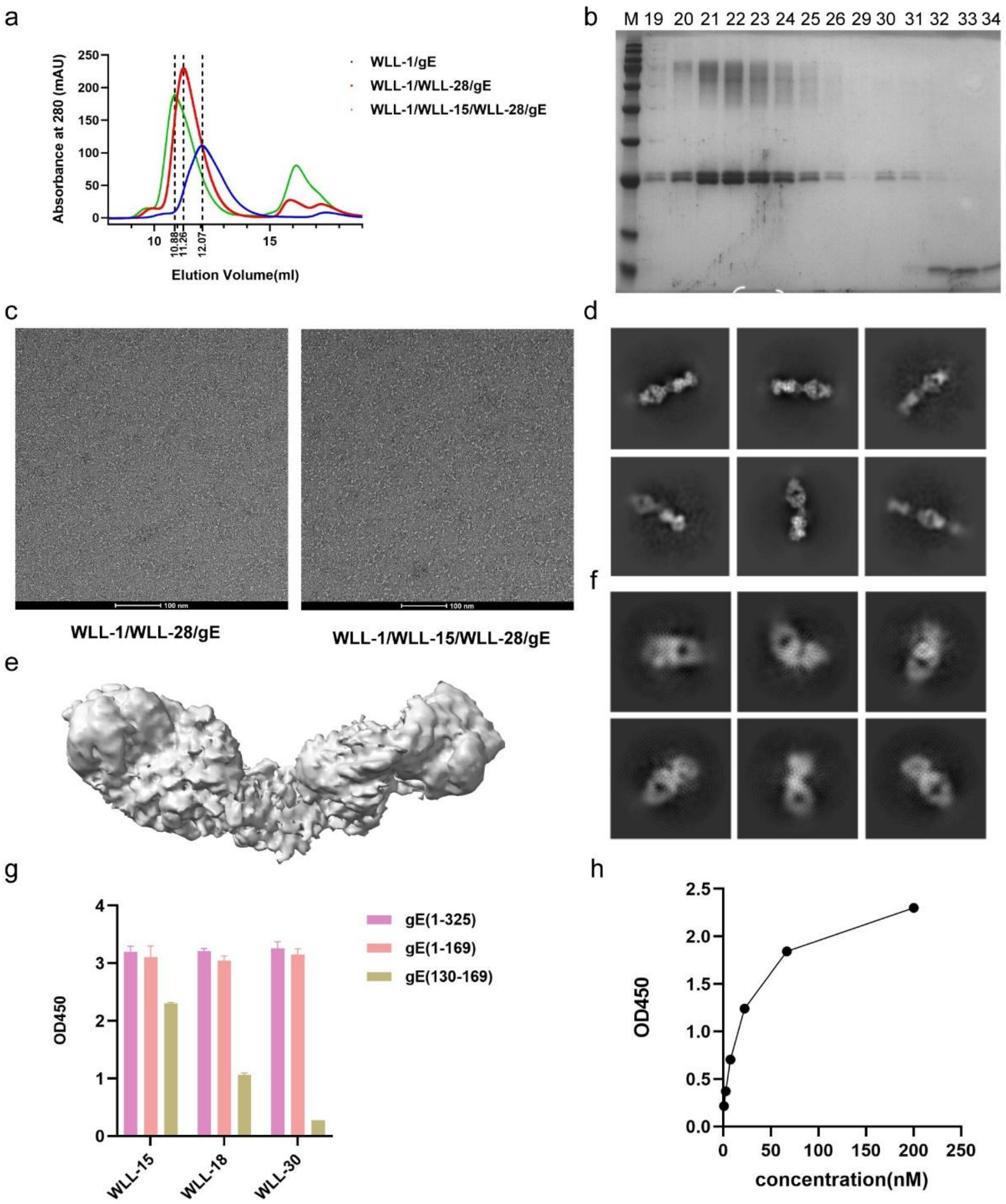

Fig. S5. Preparation and identification of samples. (**a**) Chromatographic purification profiles of different complexes. The green peak at 10.88 ml represents the Fab WLL-1/Fab WLL-15/Fab WLL-28/gE complex, the red peak at 11.26 ml represents the Fab WLL-1/Fab WLL-28/gE complex, and the blue peak at 12.07 ml represents the Fab WLL-1/gE complex. (**b**) SDS–PAGE was used to verify the purity of the complex. (**c**) The dispersion of the complex was confirmed by negative-stain electron microscopy. (**d**) 2D classification of the Fab WLL-1/WLL-15/WLL-28/gE complex. (**e**) Reconstructed 3D model of the Fab WLL-1/Fab WLL-15/Fab WLL-28/gE complex. (**f**) 2D classification of the Fab WLL-15/gE complex. (**g**) ELISA was performed to evaluate the reactivity of each antibody with various gE truncations. (**h**) ELISA of

WLL-15 at different dilutions with the 130-169 truncation. The initial concentration of the WLL-15 antibody was 200 nM, and the antibody was diluted three-fold.

Figure S6

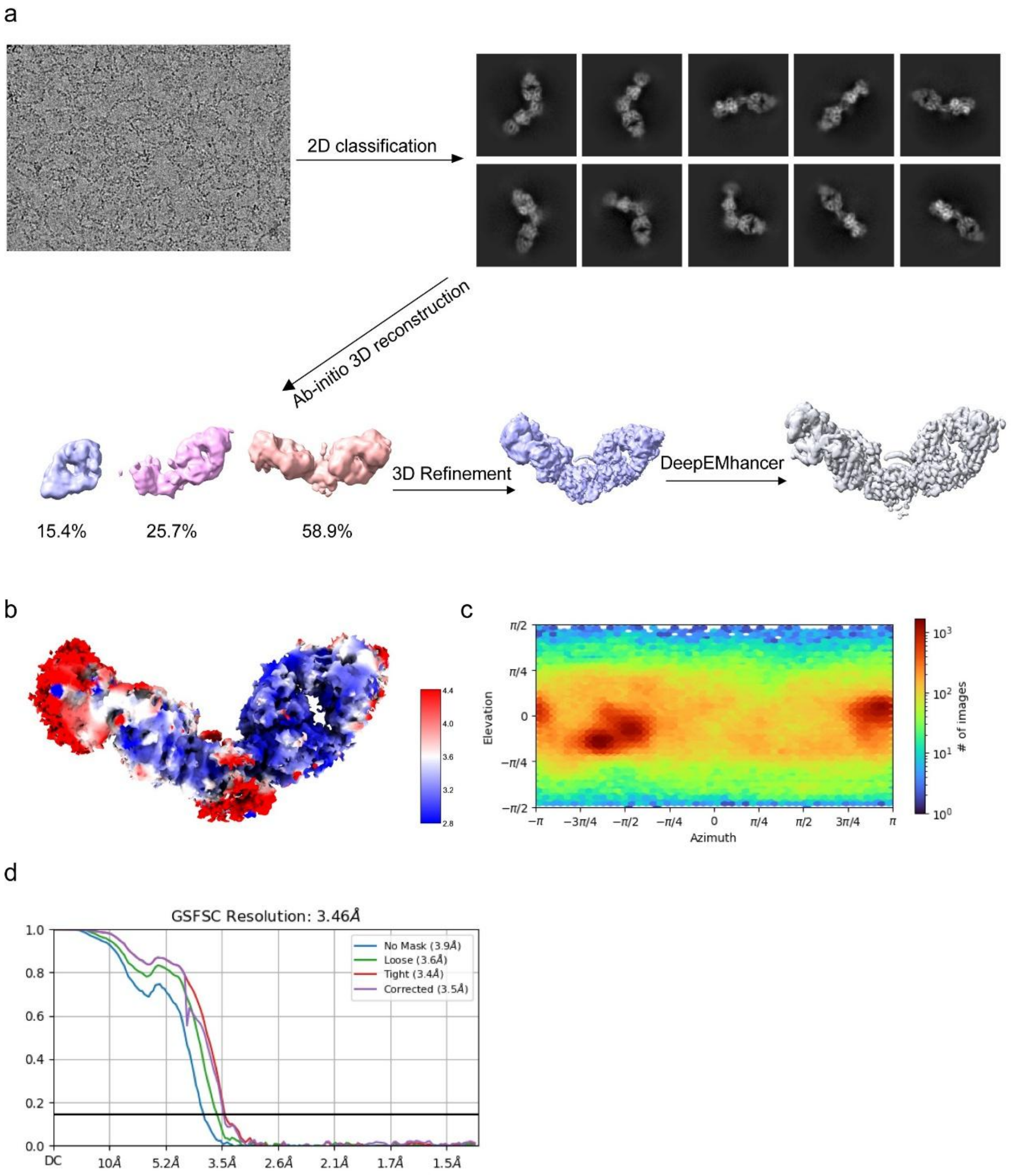

Fig. S6. Cryo-EM processing of the Fab WLL-1/Fab WLL-28/gE complex. (**a**) A total of 4,865 movies were collected, and the data processing workflow was as follows. (**b**) Isosurface rendering of the Fab WLL-1/Fab WLL-28/gE 3D map before focused refinement with surface coloring according to the local resolution estimated by windowed FSCs. (**c**) Viewing distribution plot. (**d**) Gold standard FSC curve.

Figure S7

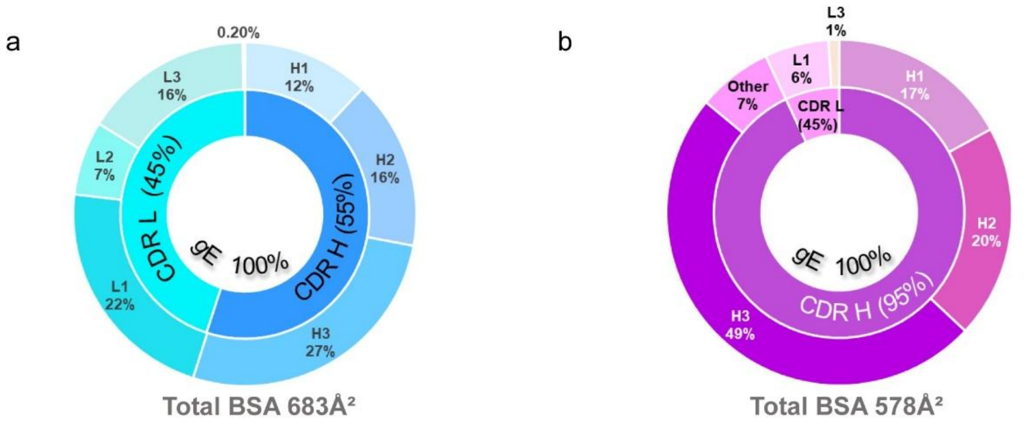

Fig. S7. Epitope detail analysis of ternary complexes. (a) The percentage contribution of Fab WLL-1 CDRs to the BSA is shown in the pie chart representation. **(b)** The percentage contribution of Fab WLL-28 CDRs to the BSA is shown in the pie chart representation.

Figure S8

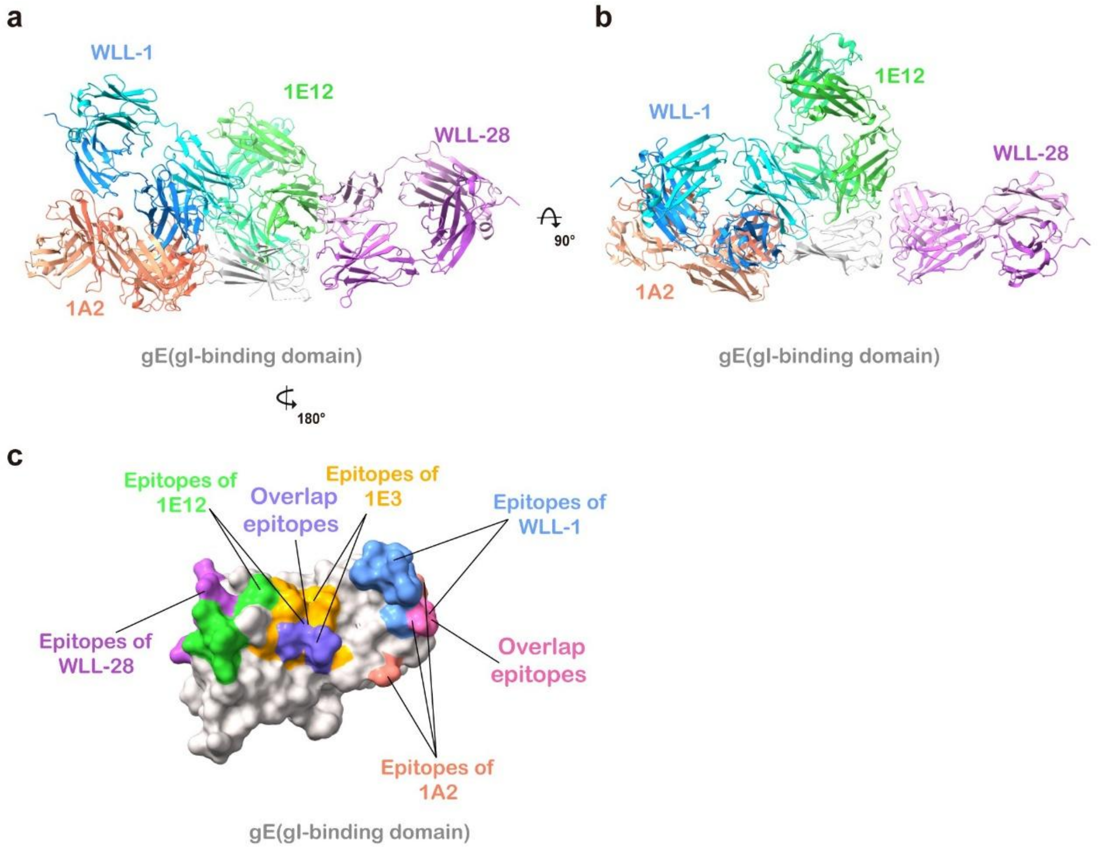

Fig. S8. Epitope analysis of WLL-1 and WLL-28 in comparison with other anti-gE antibodies from vaccine recipients. (a,b) Superimposition of antibodies WLL-1, WLL-28, 1A2 and 1E12 (PDB: 8V5L) onto the gI-binding domain. WLL-1, WLL-28, 1A2, 1E12 and the gI-binding domain are shown in cartoon representation. The gI-binding domain, WLL-1 heavy chain, WLL-1 light chain, WLL-28 heavy chain, WLL-28 light chain, 1A2 heavy chain, 1A2 light chain, 1E12 heavy chain and 1E12 light chain are gray, dodger blue, dark turquoise, medium orchid, plum, tomato, light salmon, lime green, and spring green, respectively. **(c)** Representation of the binding epitopes for anti-gE antibodies. The gI-binding domain is displayed in surface representation. The epitopes of WLL-1, WLL-28, 1E3, 1A2 and 1E12 are highlighted in cornflower blue, medium orchid, orange, salmon, and lime green. The overlapping epitopes of WLL-1 and 1A2 are colored hot pink, and the overlapping epitopes of 1E3 and 1E12 are medium slate blue.

Supplementary Tables

Table S1. Cryo-EM statistics for data collection, image processing and model building

PDB entry	9JZQ
EMDB entry	EMD-61939
Data collection and processing	
Magnification	105k
Voltage (keV)	300
Electron exposure (e-/Å^2)	60
Defocus range (μm)	-2.2 to -0.8
Pixel size (Å)	0.69
Symmetry imposed	C1
initial particle images(no.)	2,156,098
Final particle images(no.)	324,054
Map global resolution (Å)	3.48
Global resolution FSC threshold	0.143
Refinement	
Model resolution (Å)	3.48
FSC threshold	0.143
Map sharpening B factor (Å)	-151.4
Model composition	
Non-hydrogen atoms	7,890
Protein residues	1,033
Ligands	0
B factors (Å^2)	
Protein (min/max/mean)	19.32/215.27/97.12
R.m.s. deviations	
Bond lengths (Å)	0.003
Bond angles (°)	0.593
Validation	
Molprobity score	1.78
Clash score	9.10
Ramachandran plot (%)	
Outliners	0.00
Allowed	4.30
Favored	95.70

Table S2. PISA analysis of interaction between Fab WLL-1/gE and Fab WLL-28/gE

WLL-1 (683 Å)	CDR H (55%)	H1	12%
		H2	16%
		H3	27%
	CDR L (45%)	L1	22%
		L2	7%
		L3	16%
		Other	0.2%
WLL-28 (578 Å)	CDR H (93%)	H1	17%
		H2	20%
		H3	49%
		Other	7%
	CDR L (7%)	L1	6%
		L3	1%

Table S3. List of residues interacting between Fab WLL-1 and gE

	**WLL-1**	**gE**	**Distance(Å)**
Hydrogen bonds	A:THR 32[OG1]	E:TYR 192[O]	3.55
	A:TRP 55[NE1]	E:GLY 193[O]	3.13
	A:THR 32[OG1]	E:VAL 194[N]	3.78
	A:ASP 56[OD2]	E:ARG 251[NH1]	3.18
	A:ASP 58[OD2]	E:ARG 251[NH2]	3.25
	A:LEU 102[O]	E:GLY 254[N]	3.56
	B:LYS 30[NZ]	E:ALA 258[O]	2.38
	B:LYS 30[NZ]	E:ASP 259[OD1]	3.05
	B:SER 31[N]	E:ASP 259[OD2]	3.70
	B:SER 31[OG]	E:ASP 259[OD2]	3.64
	B:ASP 95[OD2]	E:GLN 253[NE2]	3.64
	B:GLY 28[O]	E:LYS 256[NZ]	2.70
	B:LYS 30[O]	E:LYS 256[NZ]	3.84
	B:ASN 65[OD1]	E:LYS 256[NZ]	3.83
Salt bridges	A:ASP 56[OD2]	E:ARG 251[NH2]	3.91
	A:ASP 56[OD2]	E:ARG 251[NH1]	3.18
	A:ASP 58[OD2]	E:ARG 251[NH2]	3.25
	B:LYS 30[NZ]	E:ASP 259[OD1]	3.05
	B:ASP 50[OD2]	E:LYS 256[NZ]	3.71

A: Fab WLL-1 heavy chain; B: Fab WLL-1 light chain; E: VZV gE

Table S4. List of residues interacting between Fab WLL-28 and gE

	WLL-28	gE	Distance(Å)
Hydrogen bonds	C:ARG 101[NH1]	E:GLU 275[O]	3.09
	C:GLY 102[N]	E:GLU 275[O]	3.55
	C:ARG 101[NH1]	E:GLU 275[OE1]	3.76
	C:LEU 104[N]	E:GLU 292[OE1]	3.32
	C:GLY 102[O]	E:GLN 294[NE2]	3.12
	D:ASP 32[OD2]	E:ARG 290[NH2]	3.39
Salt bridges	C:ARG 101[NH1]	E:GLU 275[OE1]	3.76
	C:ASP 57[OD1]	E:LYS 293[NZ]	3.39
	C:ASP 57[OD2]	E:LYS 293[NZ]	3.16
	D:ASP 32[OD2]	E:ARG 290[NH2]	3.39

C: Fab WLL-28 heavy chain; D: Fab WLL-28 light chain; E: VZV gE

Table S5. PISA analysis of gE residues interacting with Fab WLL-1

	gE residues	Bond type	Accessible Surface Area (Å^2)	Buried Surface Area (Å^2)	Solvation energy effect, kcal/mol
WLL-1 heavy chain	E:ILE 191		105.92	9.11	0.15
	E:TYR 192	H	151.53	84.64	0.89
	E:GLY 193	H	47.07	46.95	0.22
	E:VAL 194	H	21.46	12.94	0.21
	E:ARG 195		124.00	12.17	-0.13
	E:ARG 251	HS	102.82	84.76	-1.54
	E:PHE 252		31.08	1.84	-0.02
	E:GLN 253		79.74	45.36	0.22
	E:GLY 254	H	47.40	35.62	0.28
	E:LYS 255		175.56	4.17	0.03
	E:ASP 259		91.06	17.32	0.06
	E:SER 306		84.81	18.26	-0.17
WLL-1 light chain	E:GLN 253	H	79.74	34.38	-0.34
	E:LYS 255		175.56	28.19	0.26
	E:LYS 256	HS	130.09	85.75	-0.86
	E:GLU 257		129.50	8.47	-0.10
	E:ALA 258	H	93.01	58.72	0.34
	E:ASP 259	HS	91.06	73.74	-0.32
	E:GLN 260		28.70	0.46	0.00
	E:PRO 261		89.23	40.83	0.65

Table S6. PISA analysis of Fab WLL-1 interacting with gE

WLL-1 residues	Bond type	Accessible Surface Area (Å^2)	Buried Surface Area (Å^2)	Solvation energy effect, kcal/mol
A:THR 32	H	93.73	57.69	0.19
A:GLU 33		52.76	24.86	-0.08
A:TYR 54		48.24	24.44	0.23
A:TRP 55	H	124.35	37.18	0.29
A:ASP 56	HS	64.21	22.11	-0.35
A:ASP 58	HS	62.07	18.96	-0.31
A:ARG 60		136.42	4.52	-0.17
A:LEU 102	H	74.74	70.56	0.59
A:ARG 103		163.73	50.21	-0.41
A:LEU 104		112.66	1.65	0.03
A:LEU 105		134.74	59.76	0.96
B:GLY 28	H	26.13	8.47	-0.10
B:SER 29		90.89	57.34	-0.04
B:LYS 30	HS	57.14	41.84	-0.70
B:SER 31	H	45.73	31.79	-0.22
B:ASP 49		78.27	26.51	-0.32
B:ASP 50	S	62.40	18.57	-0.27
B:ASN 65	H	37.76	1.23	-0.02
B:TRP 90		134.31	41.57	0.37
B:SER 92		70.34	35.17	0.27
B:ASP 95	H	96.81	27.69	-0.08

A: Fab WLL-1 heavy chain; B: Fab WLL-1 light chain

Table S7. PISA analysis of gE residues interacting with Fab WLL-28

	gE residues	Bond type	Accessible Surface Area (Å^2)	Buried Surface Area (Å^2)	Solvation energy effect, kcal/mol
WLL-28 heavy chain	E:GLU 178		84.58	39.16	-0.11
	E:ASN 179		94.95	24.54	0.04
	E:LEU 206		100.47	48.99	0.52
	E:THR 209		99.95	17.23	0.28
	E:GLU 273		125.28	21.56	-0.25
	E:LEU 274		61.79	45.17	0.35
	E:GLU 275	HS	154.88	89.93	0.04
	E:LEU 276		40.52	22.21	0.25
	E:ARG 290		177.64	43.28	-0.16
	E:THR 291		5.58	2.57	-0.03
	E:GLU 292	H	67.13	55.77	-0.21
	E:LYS 293	S	122.49	85.60	-0.71
	E:GLN 294	H	54.24	43.75	-0.32
WLL-28 light chain	E:ASN 179		94.95	0.58	-0.01
	E:ARG 290	HS	177.64	39.78	-0.77

Table S8. PISA analysis of Fab WLL-28 interacting with gE

WLL-28 residues	Bond type	Accessible Surface Area (Å^2)	Buried Surface Area (Å^2)	Solvation energy effect, kcal/mol
C:ASN 28		95.01	7.43	-0.02
C:ALA 30		27.07	0.66	0.01
C:ASN 31		72.30	63.03	0.24
C:PHE 32		39.36	28.89	0.46
C:TRP 33		21.94	18.18	0.19
C:PHE 52		39.59	39.43	0.63
C:ALA 54		43.49	11.17	0.17
C:ASP 55		108.69	45.14	-0.53
C:ASP 57	S	44.81	17.87	0.41
C:ARG 59		121.31	19.78	-0.61
C:ARG 98		14.28	5.77	-0.21
C:VAL 100		82.99	4.78	-0.05
C:ARG 101	HS	183.45	56.25	-0.29
C:GLY 102	H	48.62	48.32	0.06
C:SER 103		49.67	25.10	0.36
C:LEU 104	H	63.11	23.31	0.27
C:TRP 105		219.60	121.56	1.80
D:ASN 30		90.30	6.59	-0.07
D:ASP 32	HS	43.41	29.41	-0.30
D:ASN 92		56.64	3.07	-0.05

C: Fab WLL-28 heavy chain; D: Fab WLL-28 light chain

References

Abramson, J., Adler, J., Dunger, J., Evans, R., Green, T., Pritzel, A., Ronneberger, O., Willmore, L., Ballard, A.J., Bambrick, J., *et al.* (2024). Addendum: Accurate structure prediction of biomolecular interactions with AlphaFold 3. Nature.

Adams, P.D., Afonine, P.V., Bunkóczi, G., Chen, V.B., Davis, I.W., Echols, N., Headd, J.J., Hung, L.W., Kapral, G.J., Grosse-Kunstleve, R.W., *et al.* (2010). PHENIX: a comprehensive Python-based system for macromolecular structure solution. Acta Crystallogr D Biol Crystallogr 66, 213-221.

Emsley, P., Lohkamp, B., Scott, W.G., and Cowtan, K. (2010). Features and development of Coot. Acta Crystallogr D Biol Crystallogr 66, 486-501.

Guo, Y., Zhang, G., Yang, Q., Xie, X., Lu, Y., Cheng, X., Wang, H., Liang, J., Tang, J., Gao, Y., *et al.* (2023). Discovery and characterization of potent pan-variant SARS-CoV-2 neutralizing antibodies from individuals with Omicron breakthrough infection. Nat Commun 14, 3537.

Hao, Y., Hao, S., Andersen-Nissen, E., Mauck, W.M., Zheng, S., Butler, A., Lee, M.J., Wilk, A.J., Darby, C., Zager, M., *et al.* (2021). Integrated analysis of multimodal single-cell data. Cell 184, 3573-3587.e3529.

McGinnis, C.S., Murrow, L.M., and Gartner, Z.J. (2019). DoubletFinder: Doublet Detection in Single-Cell RNA Sequencing Data Using Artificial Nearest Neighbors. Cell Syst 8, 329-337.e324.

Meng, E.C., Goddard, T.D., Pettersen, E.F., Couch, G.S., Pearson, Z.J., Morris, J.H., and Ferrin, T.E. (2023). UCSF ChimeraX: Tools for structure building and analysis. Protein Sci 32, e4792.

Punjani, A., Rubinstein, J.L., Fleet, D.J., and Brubaker, M.A. (2017). cryoSPARC: algorithms for rapid unsupervised cryo-EM structure determination. Nat Methods 14, 290-296.

Sanchez-Garcia, R., Gomez-Blanco, J., Cuervo, A., Carazo, J.M., Sorzano, C.O.S., and Vargas, J. (2021). DeepEMhancer: a deep learning solution for cryo-EM volume post-processing. Commun Biol 4, 874.

Ye, J., Ma, N., Madden, T.L., and Ostell, J.M. (2013). IgBLAST: an immunoglobulin variable domain sequence analysis tool. Nucleic Acids Res 41, W34-40.
